# Wenzhou TE: A First-Principle-Calculated Thermoelectric Materials Database

**DOI:** 10.3390/ma17102200

**Published:** 2024-05-08

**Authors:** Ying Fang, Hezhu Shao

**Affiliations:** School of Electrical and Electronic Engineering, Wenzhou University, Wenzhou 325035, China

**Keywords:** thermoelectric materials, material databases, high-throughput computing

## Abstract

Since the implementation of the Materials Genome Project by the Obama administration in the United States, the development of various computational materials’ databases has fundamentally expanded the choice of industries such as materials and energy. In the field of thermoelectric materials, the thermoelectric figure of merit (ZT) quantifies the performance of the material. From the viewpoint of calculations for vast materials, the ZT values are not easily obtained due to their computational complexity. Here, we show how to build a database of thermoelectric materials based on first-principle calculations for the electronic and heat transport of materials. Firstly, the initial structures are classified according to the values of bandgap and other basic properties using the clustering algorithm K-means in machine learning, and high-throughput first principle calculations are carried out for narrow-bandgap semiconductors which exhibit a potential thermoelectric application. The present framework of calculations mainly includes a deformation potential module, an electrical transport performance module, a mechanical and a thermodynamic properties module. We have also set up a search webpage for the calculated database of thermoelectric materials, providing search facilities and the ability to view the related physical properties of materials. Our work may inspire the construction of more computational databases of first-principle thermoelectric materials and accelerate research progress in the field of thermoelectrics.

## 1. Introduction

In 2011, the Obama administration of the United States officially proposed the “Material Genome Project”, which utilized high-throughput computing and experiments to obtain massive quantities of material data, combined with data analysis technology by artificial intelligence for new material development. The goal was to shorten the cycle of new material development and applications, as well as reduce the costs for material research and development, so that the United States could continue to maintain its leading position in manufacturing technology. In 2016, the US government released the “First Five Years of the Materials Genome Initiative: Accommodations and Technical Highlights” report, which pointed out that during the five years of the implementation of the Materials Genome Engineering program, federal research institutions such as the Department of Energy, the Department of Defense, the Natural Science Foundation, the National Bureau of Standards and Technology, and the National Aeronautics and Space Administration had invested over USD 500 million, establishing computational material research and development centers including the National Network for Virtual High throughput Preparation (NIST&NREL) and the Center for Cross Scale Material Design and Multi Scale Materials Research (NIST, ANL, ARL), forming three major computational material databases: the Materials Project (MP) [1], AFLOW [2], and OQMD [3,4]; several auxiliary databases such as the Materials Data Repository (MDR), the Materials Resource Registry, and the Energy Materials Network, as well as database-related analytical tools.

Shortly after the proposal of the Materials Genome Project by the United States, the European Science Foundation launched the Accelerated Metallurgy (ACCMET) program, which cost over EUR 2 billion, with the aim of keeping up with the pace of the United States. The European Commission funded the Horizon 2020 project NoMaD, led by the Max Planck Institute in Germany, for a period of three years in 2015. The project aimed to use the “centralized data warehouse” method to involve various research groups and provide data related to computational materials science, with the aim of building a “Encyclopedia of Materials” and a tool for analyzing big data on materials. In the UK, the government has also implemented an e-science program, with funding to carry out high-throughput material computing simulations and the construction of material computing basic databases, such as eMinerals and the “Material Grid” project. The Swiss EPFL University has led the development of the European materials database, AiiDA [5].

Nowadays, with the vigorous development of big data and artificial intelligence technology, the material genome project research characterized by high-throughput experiments, high-throughput computing, and artificial intelligence big data analysis is in full swing, and has shown astonishing advantages in many materials’ fields. The paper “Machine-learning-assisted materials discovery using failed experiments”, published in Nature in May 2016 [6], showed that based on years of accumulated experimental data, various catalytic new materials could be discovered using artificial intelligence (AI) technology. This work indicated that AI will profoundly transform the research methods in the field of materials. The centuries long history of human scientific development has formed three research paradigms: experimental, theoretical, and computational. However, in the fields of complex systems such as biology, astronomy, and materials, there are very complex interactions involved, coupled with a large number of variables, which greatly limits the effectiveness of theoretical and computational research models and requires the combination of big data and AI as the “fourth paradigm”. In 2017, AlphaGo defeated the human Go master of the boardgame, but Google disbanded the DeepMind team responsible for developing the program, and then formed an AI research and development team engaged in material genome engineering. At present, American high-tech companies including Apple, Google, IBM, Tesla, etc., are all laying out the use of AI for the research and development of new materials based on material genomic methods. The fourth paradigm of materials science requires the ability to generate and process massive amounts of data, thus obtaining massive amounts of material data has become a key aspect of the Materials Genome Project. With the improvement of computing power, the accumulation of material data based on high-throughput computing is receiving more and more attention, and its application in the research and development of new thermoelectric materials is expected to greatly accelerate its application process.

The performance of thermoelectric materials is described by the figure of merit (ZT), which can be expressed as follows:

(1) $$ZT=\frac{S^{2}\sigma T}{\kappa_{e}+\kappa_{l}}$$

where S is the Seebeck coefficient; σ is the conductivity; T is the temperature, $\kappa_{e}$; and $\kappa_{l}$ is the thermal conductivity contributed by carriers and phonons, respectively. These parameters of S, σ, and κ are coupled with each other, and it is difficult to independently regulate them. For example, for semiconductor materials, increasing doping concentration can increase conductivity, while at the same time reducing the Seebeck coefficient and increasing the carrier thermal conductivity. At present, the three major material databases, Materials Project, AFLOW, and OQMD, have data on several common physical quantities, including atomic and band structure, and other physical properties are also being added. However, the thermoelectric performance of materials, due to their particularity and the complexity in calculating electrical and thermal transport properties, generally require a large amount of computation.

Here, we have selected the Materials Project as the structural source for constructing a thermoelectric material database. Specifically, we employed the atomic structure files POSCAR and CIF (currently 19952 materials) in MP materials with an id number below 100,000 through the Materials Project API as the initial materials for building the present thermoelectric material database—**Wenzhou TE**. We built deformation potential modules, mechanical properties (elastic properties) modules, and electronic transport using BoltzTraP modules. And then, we collected data via Python scripts and displayed them on a web site, https://hezhu2024.github.io (accessed on 3 April 2024), for others to use.

## 2. Methodology

### 2.1. Clustering (K-Means)

At present, the excellent thermoelectric materials obtained in experiments are mainly semiconductors with narrow bandgaps; then, we chose the bandgap as a major feature for material screening. At the same time, we selected free energy, volume, density, and average atomic energy as the other features from the descriptors obtained from the MP database. They form the five featured variables for the K-means clustering algorithm.

Here is a brief introduction to the K-means principle [7]. K-means clustering is a non-hierarchical supervised pattern recognition method where a pre-defined numbers of clusters are formed. It divides the data into K classes. Firstly, K class random points are randomly generated, denoted as $O_{1},O_{2},{\cdots O}_{l},\cdots O_{K}$. Assuming that the *j*-th feature of the *i*-th data is represented as $x_{ij}$, the distance from the i-th data sample to the *l*-th class random point is:

(2) $$d_{il}=\sqrt{\sum_{j=0}^{j=J}{\left(x_{ij}-O_{lj}\right)}^{2}}$$

among them, *J* represents a total of *J* features in the data. The random class point with the smallest distance represents the same class. After the first iteration, each data sample will be classified into a certain class. Then, we calculated the average value of each class of data as the new random class point. The new random class point can be represented as:

(3) $$O_{lj}=\frac{1}{N}\sum_{i=0}^{i=N}x_{ij}$$

among them, $j\in \left[1,2,\ldots ,J\right],l\in [1,2,\ldots ,K]$.

Then, we re-calculated these distances, and reclassified them, and this process was repeated until convergence was achieved. And finally, the data were classified into K classes. In the present work, we also standardized the data before classification. In order to illustrate how many categories were most reasonable, we could assume that the formula for the total loss was as follows:

(4) $$Loss=\sum_{i=0}^{i=n}d_{il}$$

where *n* represents the number of samples. This formula represented the sum of distances from all sample points to their random class points. When there was a significant inflection point on the line of loss with respect to class K, the value of K at the inflection point should be considered as a reasonable classification. Through the K-means method, we divided the initial materials from MP into five categories. Their quantities were 6602, 5425, 3770, 2800, and 1355, respectively.

### 2.2. Deformation Potential Theory (DPT)

The deformation potential theory was proposed by Bardeen and Shockley [8] in the 1950s to describe the charge transfer in non-polar semiconductors. The charge mobility can be expressed as *μ_x_* = *eτ_x_*/m*, where the relaxation time for bulk materials could be written as follows [8,9]:

(5) $$\tau_{x}=\frac{2\sqrt{2\pi }\hbar^{4}C_{x}}{3{\left(k_{B}Tm*\right)}^{\frac{3}{2}}E_{DPx}^{2}}$$

where $C_{x}=\partial^{2}E/({\partial (∆a_{x}/a_{x})}^{2}V_{0})$ is the elastic constant; $E_{DPx}=∆V_{i}/(∆a_{x}/a_{x})$, $∆V_{i}$ is the deformation potential energy, which is the difference between the energy level of the i-th energy band and the energy level of the deep nuclear state; and m* = $\hbar^{2}/(\partial^{2}E/{\partial k}^{2})$ is the effective mass.

### 2.3. Elastic and Thermal Properties

We can obtain the elastic properties, group velocity, Poisson’s ratio, Debye temperature, Grüneisen coefficients, and lattice thermal conductivity, after calculating the elastic constants of materials [10], which could be easily achieved during the high-throughput calculations.

In the case of uniform deformation for a crystal, the generalized form of Hooke’s law of stress–strain [11] is:

(6) $$f_{ij}=C_{ijkl}\epsilon_{kl}$$

where $f_{ij}$ and $\epsilon_{kl}$ is a homogeneous second-order stress tensor and a strain tensor, respectively [12]. $C_{ijkl}$ represents the fourth order elastic stiffness tensor. Using matrix representation, we can abbreviate the stiffness tensor $C_{ijkl}$ of four suffixes to the stiffness tensor $C_{ij}$ of two suffixes, which can be represented as follows:

(7) $$C_{ij}=\left[\begin{array}{c} \begin{array}{cc} C_{11} & C_{12}\\ C_{21} & C_{22} \end{array}\;\;\;\;\begin{array}{cc} C_{13} & C_{14}\\ C_{23} & C_{24} \end{array}\;\;\;\;\begin{array}{cc} C_{15} & C_{16}\\ C_{25} & C_{26} \end{array}\\ \begin{array}{cc} C_{31} & C_{32}\\ C_{41} & C_{42} \end{array}\;\;\;\;\begin{array}{cc} C_{33} & C_{34}\\ C_{43} & C_{44} \end{array}\;\;\;\;\begin{array}{cc} C_{35} & C_{36}\\ C_{45} & C_{46} \end{array}\\ \begin{array}{cc} C_{51} & C_{52}\\ C_{61} & C_{62} \end{array}\;\;\;\;\begin{array}{cc} C_{53} & C_{54}\\ C_{63} & C_{64} \end{array}\;\;\;\;\begin{array}{cc} C_{55} & C_{56}\\ C_{65} & C_{66} \end{array} \end{array}\right]$$

The elastic flexibility tensor $\left(s_{ij}=C_{ij}^{-1}\right)$ can be written as:

(8) $$s_{ij}=\left[\begin{array}{c} \begin{array}{cc} s_{11} & s_{12}\\ s_{21} & s_{22} \end{array}\;\;\;\;\begin{array}{cc} s_{13} & s_{14}\\ s_{23} & s_{24} \end{array}\;\;\;\;\begin{array}{cc} s_{15} & s_{16}\\ s_{25} & s_{26} \end{array}\\ \begin{array}{cc} s_{31} & s_{32}\\ s_{41} & s_{42} \end{array}\;\;\;\;\begin{array}{cc} s_{33} & s_{34}\\ s_{43} & s_{44} \end{array}\;\;\;\;\begin{array}{cc} s_{35} & s_{36}\\ s_{45} & s_{46} \end{array}\\ \begin{array}{cc} s_{51} & s_{52}\\ s_{61} & s_{62} \end{array}\;\;\;\;\begin{array}{cc} s_{53} & s_{54}\\ s_{63} & s_{64} \end{array}\;\;\;\;\begin{array}{cc} s_{55} & s_{56}\\ s_{65} & s_{66} \end{array} \end{array}\right]$$

The Voigt [13] bulk modules can be calculated by:

(9) $$B_{v}=\frac{1}{9}\left[\left(C_{11}+C_{22}+C_{33}\right)+2\left(C_{12}+C_{23}+C_{31}\right)\right]$$

The shear modulus can be obtained by:

(10) $$G_{v}=\frac{1}{15}\left[\left(C_{11}+C_{22}+C_{33}\right)-\left(C_{12}+C_{23}+C_{31}\right)+3\left(C_{44}+C_{55}+C_{66}\right)\right]$$

The Reuss [14] bulk and shear modulus can be calculated by,

(11) $$\frac{1}{B_{r}}=\left(s_{11}+s_{22}+s_{33}\right)+2\left(s_{12}+s_{23}+s_{31}\right)$$

and

(12) $$\frac{15}{G_{r}}=4\left(s_{11}+s_{22}+s_{33}\right)-4\left(s_{12}+s_{23}+s_{31}\right)+3\left(s_{44}+s_{55}+s_{66}\right)$$

In the present work, we took the arithmetic mean of the boundaries between Voigt and Reuss, Voigt–Reuss–Hill (VRH) [14]:

(13) $$B_{h}=\frac{B_{r}+B_{v}}{2}$$

(14) $$G_{h}=\frac{G_{r}+G_{v}}{2}$$

The longitudinal ($v_{l}$), transverse ($v_{t}$), and average ($v_{a}$) elastic wave velocities can be calculated by:

(15) $$v_{l}=\sqrt{\frac{3B_{h}+4G_{h}}{3\rho }},$$

(16) $$v_{t}=\sqrt{\frac{G_{h}}{\rho }},$$

(17) $$v_{a}={\left[\frac{1}{3}\left(\frac{2}{v_{t}^{3}}+\frac{1}{v_{l}^{3}}\right)\right]}^{-1/3}$$

The Debye temperature ($\theta_{D}$) was obtained by:

(18) $$\theta_{D}=\frac{h}{k_{B}}{\left[\frac{3q}{4\pi }\frac{N\rho }{M}\right]}^{1/3}v_{a}$$

And the Grüneisen coefficient was calculated by:

(19) $$\gamma =\frac{3}{2}\left(\frac{1+v_{poi}}{2-3v_{poi}}\right)$$

where $v_{poi}=(1-2{\left(\frac{v_{t}}{v_{l}}\right)}^{2})/(2-2{\left(\frac{v_{t}}{v_{l}}\right)}^{2})$ is the Poisson’s ratio.

According to the Slack formula [15,16], the lattice thermal conductivity can be expressed as:

(20) $$k_{l}=A\frac{\bar{M}\theta_{D}^{3}\delta }{\gamma^{2}n^{2/3}T}$$

where $\bar{M}$ is the average atomic mass; $\theta_{D}$ is the Debye temperature; δ is the volume of each atom; n is the number of atoms in the original cell; γ is the Grüneisen coefficient; A is a constant of $3.1\times 10^{-6}$; and *T* is the temperature.

### 2.4. Methods for the First-Principles Calculations and Transport Properties

In the process of building a thermoelectric material database, first-principles calculations were performed using the Vienna Ab initio Simulation Package (VASP) 5.4.1 [17,18]. The generalized gradient approximation (GGA) with Perdew, Burke, and Ernzerhof functional (PBE) was employed [19]. The calculation of electronic transport required the use of the BoltzTraP2 program package [20]. In order to minimize computational costs while ensuring data reliability, during optimizing calculations, we set the plane-wave energy cutoff to be 1.4 times the maximum ENMAX of POTCAR of the composed elements, the electronic energy convergence to be 10^−4^ eV, the force convergence for ions to be 10^−2^ eV/Å, and the density k-mesh to be 0.04 × 2π Å^−1^.

All the processes were controlled through shell scripts. Data collection and calculation were implemented using Python 3 scripts. These codes were all home-made.

## 3. Capabilities and Workflow

### 3.1. The Application of K-Means on Datasets from MP

**Figure 1:**
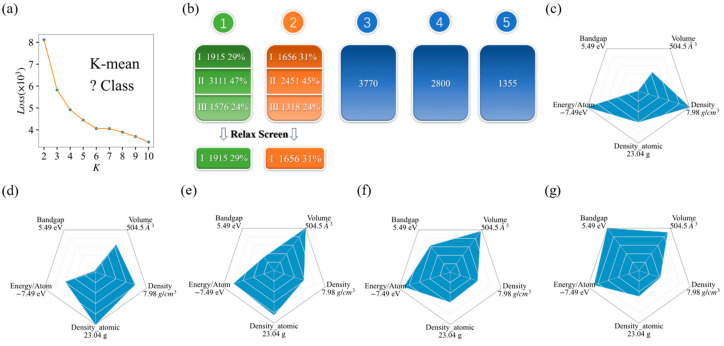
The application of K-means on MP databases: (**a**) the line chart of loss for K-class; (**b**) the classification data and relaxation screening results of the initial structures under K-means; (**c**–**g**) the average distribution of 5 features for each K-means class.

From Figure 1a, it can be seen that the number of points with an obvious inflection is six, which means that the initial structures can be divided into six categories. Considering the reasonable distribution of the average-bandgap values, we ultimately divided it into five categories. The featured distribution map and various information of K-means are shown in Figure 1c–g. The average value of bandgap for the first class is merely 0.025 eV, so this class of material contains many metals. The second class with an average bandgap value of 0.14 eV was mainly composed of semiconductors with narrow bandgaps. The third, fourth, and fifth categories were mainly composed of semiconductors and insulators with wide bandgaps. As a starting point, we focused on calculating the physical properties of candidate material sets for the first and second categories.

**Figure 2:**
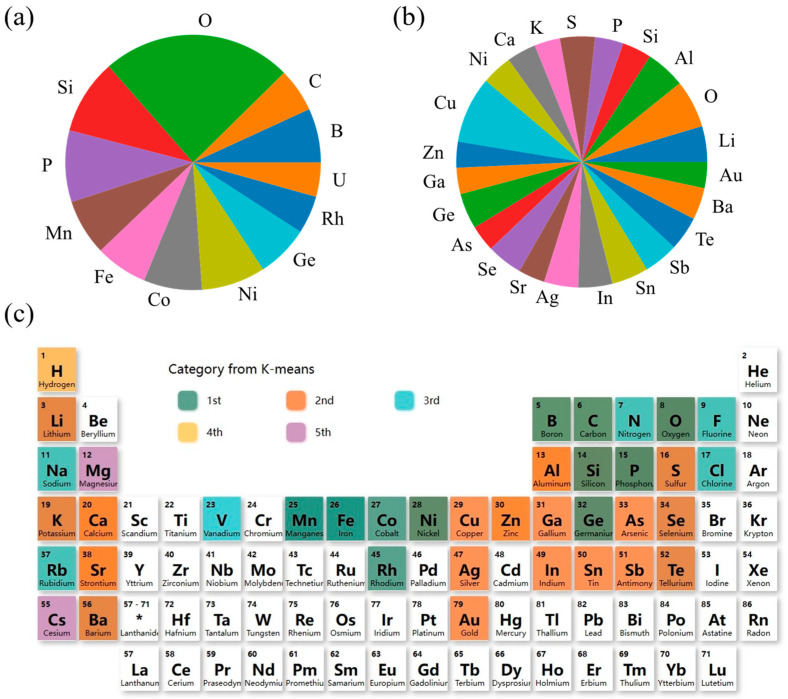
Element statistics for 5 classes of materials: (**a**) pie charts of elements for the first class; (**b**) pie charts of elements for the second class; (**c**) distribution charts of elements for 5 classes, and * represents for the rare-earth metal.

The material data collected from the MP database are finally divided into five categories using the K-means method. We identified the elements which are contained in the compounds of every class, and selected those larger than 5% of the total materials in number. For instance, in the first class, there are 6602 materials, if some element contained more than 330 (6602 × 5%) materials, we picked it out. As shown in Figure 2a, the first class of materials are mainly contributed by O, Si, P, Mn, Fe, Co, Ni, Ge, Rh, U, B, C, etc. In the second class, the materials are mainly composed of the III, IV, V, and VI main groups, with the alkali and alkaline-earth metals. In the sets of elements in the first and especially second class, the main group elements of III, IV, V, and VI are the habitats of many known semiconductors, suggesting the possible existence of excellent thermoelectric materials. Compared with the first and second class, many compounds in the third one are contributed by the halogen elements (such as F, Cl), and many of them take perovskite structures (such as CsPbCl_3_, CsGeCl_3_, etc.). The appearance of the H element in the fourth category indicates the presence of many hydrides and even organic compounds. And the fifth category has added Cs and Mg elements, most of which have larger bandgaps. By using the K-means method to classify the raw data for the first time, we can select the material dataset that needs to be calculated, which could help to reduce the calculation time.

### 3.2. Computational Framework and Relaxation Process

After obtaining the structural file, we firstly performed structural relaxation and static calculation. Structural relaxation refers to the optimization process of atomic positions and lattice constants. We have employed VASP 5.4.1 for the first-principle calculations. Actually, several mainstream databases such as AFLOW, MP, OQMD, etc., were also calculated using VASP software.

**Figure 3:**
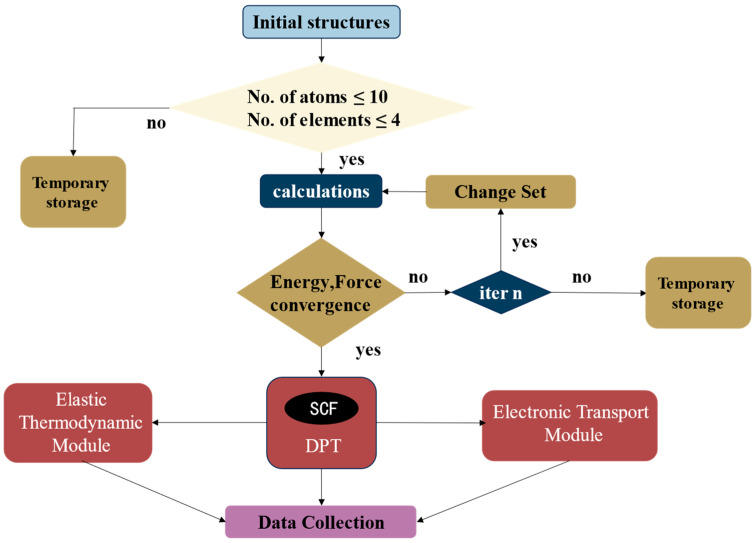
Process flowchart for constructing thermoelectric material database.

For the first and second classes of materials obtained through K-means initial screening, there were more than 12,000 materials, many of which contained too many element classes and numbers of atoms in the primitive cell. In the present work, we firstly calculated the material system with a relatively simple structure. Therefore, a computational control process was employed during the structural relaxation to further screen them, resulting in a total of more than 3000 materials with relatively simple structures in the first and second classes. Nevertheless, conducting structural relaxation for so many materials was a computationally demanding task. In order to accelerate the calculation, we wrote several shell scripts to control the process of structural relaxation. The flowchart is shown in Figure 3.

After performing relaxation calculations on the data of the first and second classes of materials, we screened 1915 and 1656 materials, respectively, for further calculations, as shown in Figure 1b. In the first class, there are 3111 materials remaining with atomic numbers greater than 10 or element classes greater than 4, and the other 1576 materials are unrelaxed structures which are hard to obtain convergent relaxation for in our present setup calculations. In the second category, there are also 2451 materials with atomic numbers greater than 10 or element classes greater than 4, and 1318 materials that are difficult to be relaxed. After the relaxation calculation process, the convergent structures are saved for further calculations.

Then, we performed the calculations of the parameters of deformation potential theory. Firstly, we performed an anisotropic property judgment on the material, and then we performed static calculations with a density of 0.04 × 2π Å^−1^ k-mesh set on the deformed structures in various directions.

### 3.3. Analysis of Results of Deformation Potential Theory (Using Si as an Example)

**Figure 4:**
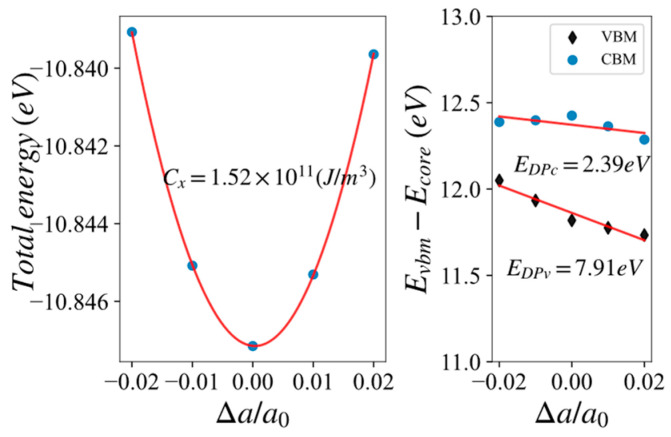
Schematic diagram of second-order fitting elastic constant $C_{x}$ and first-order fitting of elastic potential energy $E_{DP}$ for Si.

The deformation potential method considered acoustic phonons as the main scattering sources for electrons. The relaxation time obtained by ignoring the contributions of optical phonon branches and other scattering mechanisms could be larger than the real one, but the calculation of deformation potential is relatively simple, easily employed in high-throughput calculations. The coefficients for applying deformation to the lattice vector are {0.98, 0.99, 1.00, 1.01, 1.02} of relaxed volumes, respectively. Such calculations could ensure the reliability of fitting with the second-order function for the elastic constant and the first-order function for the elastic potential energy. We took Si as an example, as shown in Figure 4.

After calculating the deformation potential parameters, we could obtain the relaxation time of carriers by combing the effective masses.

### 3.4. Energy Band and Effective Mass Calculation

**Figure 5:**
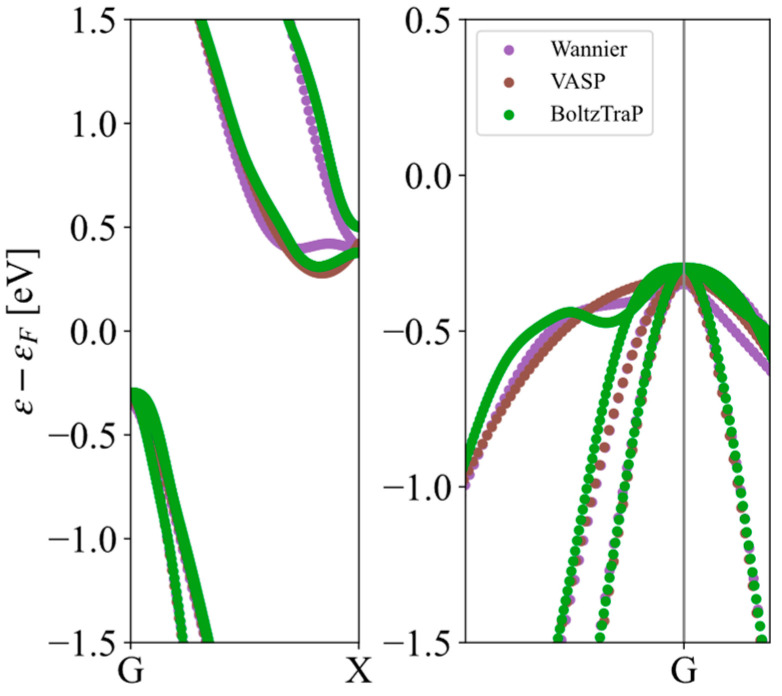
The band structure of Si by three schemes: VASP, BoltzTraP, and Wannier.

There are many methods to obtain the band structure of a material. Here, we compare three feasible schemes. The first scheme is calculating the energy band along the high symmetry point by VASP, the second one is using BoltzTraP2 [20] to fit the band structure by VASP, and the third one is using maximally localized Wannier function to interpolate the VASP results [21]. Considering the accuracy and efficiency, the second scheme was chosen in the present high-throughput calculations. The bandgap of Si in the MP database is 0.61 eV, which is consistent with VASP calculation. The relative error of the bandgap (0.59 eV) calculated by BoltzTraP is within 5%. The effective mass of Si calculated by BoltzTraP is similar to that of VASP calculation, and the relaxation time of electrons is around 1113 fs, as listed in the Table 1. The energy band of Si by three schemes is shown in Figure 5. Although set relatively coarse for the k-mesh set, the electronic properties’ calculations could also converge to some reliable results, which will be discussed in followed text, due to the good fitting for the energy at the high symmetry point. We note here that the BoltzTraP calculation for the band structure is the fastest one; then, it is suitable for accelerating the high-throughput calculation.

**Figure 6:**
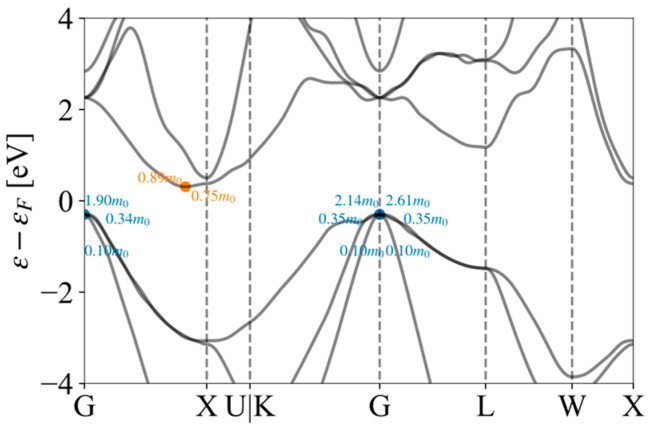
The effective mass of Si by the BoltzTraP scheme.

To facilitate the high-throughput calculation, we used the formula m* = $\hbar^{2}/(\partial^{2}E/{\partial k}^{2})$ to calculate the effective mass. The effective masses of Si by the BoltzTraP scheme are shown in Figure 6. A series of effective masses of conduction and valence bands were obtained near the high symmetry points of Γ and X. We selected the maximum values of 0.89 $m_{0}$ and 2.61 $m_{0}$ as the effective masses for the conduction band and valence band, respectively. In addition, our program was designed to automatically determine whether the band is degenerate and calculate the effective mass for each degenerate band. We note here that the reason for selecting the maximum effective mass is that the deformation potential method could overestimate the relaxation time. By selecting the maximum effective mass, the relaxation time can be effectively reduced to compensate for the shortcomings of the deformation potential theory. In high-throughput calculations, the program also selected representative effective masses for other materials such as Si.

### 3.5. High-Throughput Electrical Transport Properties (BoltzTraP)

BoltzTraP is a program package calculating the semi-classic transport coefficients, based on a smoothed Fourier interpolation of the bands. The electronic transport properties such as Seebeck coefficient, electronic conductivity, and electronic thermal conductivity can be obtained at different temperatures and different doping concentrations. The BoltzTraP program has an input interface for VASP files, which could meet the needs of presenting high-throughput processes. After completing static calculations, the BoltzTraP module can be performed. Meanwhile, BoltzTraP based on Python can be properly embedded into our high-throughput Python data processing scripts, which are written for quickly obtaining the calculated quantities such as Seebeck coefficient, electronic conductivity, and electronic thermal conductivity. Combined with the lattice thermal conductivities estimated from the elastic property calculations, we could obtain the ZT values for the materials. For semiconductors, doping concentration is set within the range from $1.0\times 10^{18}\;{cm}^{-3}$ to $1.0\times 10^{21}\;{cm}^{-3}$. During the statistic process, we left materials with a large relaxation time (>10^12^ fs) for further testifying, and it remained for renewal in the next version of the database. For some materials, it is hard to obtain a reliable value for the deformation potential energy with the current calculation set, and then obtain a large relaxation time. When such relaxation times were fed into BoltzTraP, it would lead to unreliable conductivity and electronic thermal conductivity. The choice of n-type or p-type depends on where the maximum ZT value occurs. We list the top five semiconductor materials with ZT values in Table 2.

**Figure 7:**
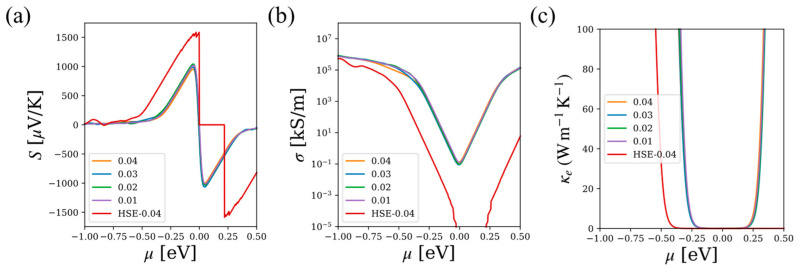
The performance of electronic transport under different k-mesh density set of PBE or HSE (0.04) at 300 K for Si: (**a**) Seebeck coefficient S; (**b**) conductivity σ; and (**c**) the electronic thermal conductivity $\kappa_{e}$ with respect to different chemical potentials μ.

To further discuss the effectiveness of the present calculations, we took Si as an example to discuss the performance of electronic transport properties under different k-mesh sets during static calculations. For Si, a density of 0.04 × 2π Å^−1^ k-mesh set corresponds to a k-point grid of 8×8×8, and that of 0.01 × 2π Å^−1^ is for a 32×32×32 k-mesh set. In BoltzTraP, the more k-mesh points provided, the more accuracy reached for the calculated electronic transport performance. As shown in Figure 7, a k-mesh density of 0.04 × 2π Å^−1^ could guarantee moderately accurate results and greatly reduces the calculation time. We also gave the HSE [23] results for comparison. The computational cost of HSE was demanding; we will provide the results using the HSE method in the next version of the database.

### 3.6. ZT Value and BE Value

**Figure 8:**
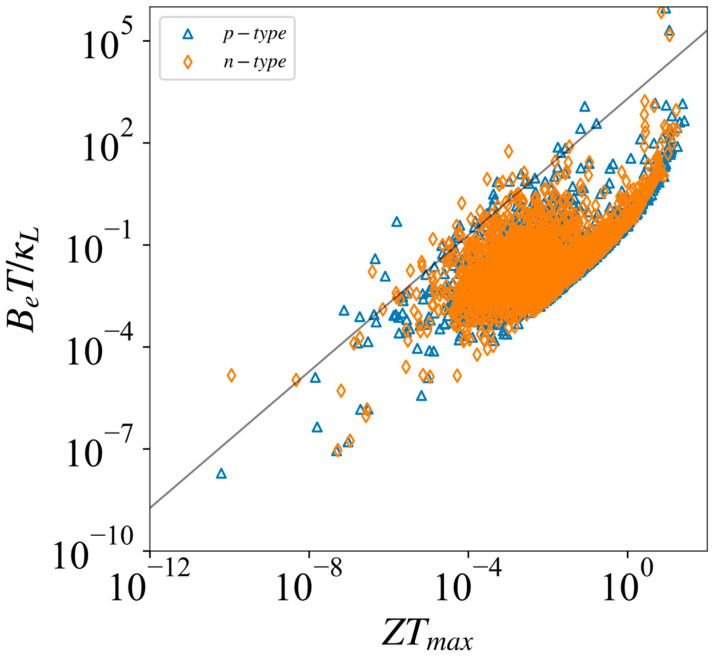
Thermal power quality factor $B_{E}T/\kappa_{L}$ and maximum ${ZT}_{max}$ at 300 K. The grey line is a linear fitting for all the *ZT* and $B_{E}T/\kappa_{L}$ values, which used to show a trend of linear positive correlation.

As an example for the application of our database, we associated the thermoelectric ZT values with the electronic quality factor of $B_{E}T/\kappa_{L}$. By S and σ, the electronic quality factor $B_{E}$ can be defined by [24]:

(21) $$B_{E}=S^{2}\sigma \left[\frac{S_{r}^{2}exp\left(2-S_{r}\right)}{1+exp\left[5-5S_{r}\right]}+\frac{S_{r}\pi^{2}/3}{1+exp\left[5\left(S_{r}-1\right)\right]}\right]$$

where $S_{r}=\left|S\right|e/k_{B}$. As shown in Figure 8, the ZT values of most materials are positively correlated to the electronic quality factor of $B_{E}T/\kappa_{L}$, so the $B_{E}T/\kappa_{L}$ values could also serve as another criterion for judging the performance of a thermoelectric material.

### 3.7. Validation and Future Development of the Database

We built a database of thermoelectric materials. We note here that the errors in our data come from the following aspects: (1) the PBE method was employed to calculate the band structures, which generally results into the underestimation for the band gap; (2) the rigid band model was used in BoltzTraP, which may fail to give the change for the band structures during doping; (3) the deformation potential model was used to obtain the electron lifetimes, which could be much higher than the experimental results, for such a model generally underestimates the effect of electron-phonon scattering; and (4) the lattice thermal conductivities were estimated from the calculated elastic coefficients, which hardly described the phonon–phonon scattering in materials.

In the next versions of the Wenzhou TE database, we will gradually improve the reliability of the data, starting with a high-throughput calculation of the thermal conductivity by accounting the three-phonon scattering. Secondly, we will use HSE method to perform high-throughput band structure calculations. We will also use some other models to improve the prediction of electronic lifetimes. And lastly in the calculation, we will add the convergence judgment.

## 4. Conclusions

In this work, we built a thermoelectric material database—Wenzhou TE. We designed several modules to obtain the electronic and heat transport parameters for materials, including structural screening, deformation potential, elastic constant, and BoltzTraP electronic transport performance calculations module. In addition, we wrote several Python scripts to collect data and process results. Furthermore, we also built a webpage for Wenzhou TE: a first-principle-calculated thermoelectric materials database (https://hezhu2024.github.io (accessed on 3 April 2024)), which could be used for searching and viewing the physical properties of materials. Subsequently, we will continue the development of the database by employing more accurate methods for electronic and phonon transport properties, and also calculating more materials. Based on the present work, one could easily use these data for data mining and thermoelectric material development.

## Figures and Tables

**Table 1:** Calculated deformation potential parameters, effective mases, relaxation time of carriers, elastic coefficients C_ij_ (in GPa), bulk modulus B (in GPa), and shear modulus G (in GPa) for Si.

Carrier Type	*E_DPx_* (eV)	*C_x_* (10^11^ Jm^−3^)	m*/*m*_0_	*τ_x_* (fs)
Electron (Hole)	2.39 (7.91)	1.5 (1.5)	0.89 (2.61)	1113 (20)
C_11_	C_12_	C_44_	B	G
153.7 (165.8 *)	57.2 (64.0 *)	74.8 (79.6 *)	89.4 (97.9 *)	62.8 (66.5 *)

* is the experimental results [22].

**Table 2:** Top 5 semiconductor materials sorted by ZT value at 300 K.

Id	Formula	*N* (cm^−3^)	*κ_l_* (WK^−1^m^−1^)	*S* (μV/K)	*σ* (kS/m)	*κ_e_* (WK^−1^m^−1^)	ZT	Type
mp-23231	AgBr	1.11 × 10^19^	0.76	433.58	187.71	1.14	5.56	P
mp-22919	AgI	5.81 × 10^18^	1.31	421.47	279.56	1.76	4.85	P
mp-27484	Tl_4_O_2_	2.69 × 10^19^	0.67	361.13	196.98	0.92	4.83	P
mp-22922	AgCl	7.05 × 10^19^	0.51	383.05	100.90	0.51	4.34	P
mp-32791	Ag_4_S_2_	1.78 × 10^19^	0.39	310.31	144.72	0.80	3.52	N

## Data Availability

Data are contained within the article.
